# Apply EZStrobe to simulate the finishing work for reducing construction process waste

**DOI:** 10.1038/s41598-023-50442-4

**Published:** 2024-01-03

**Authors:** Pham Vu Hong Son, Pham Van Tien

**Affiliations:** Faculty of Civil Engineering, Ho Chi Minh City University of Technology (HCMUT), Vietnam National University (VNU-HCM), Ho Chi Minh City, Vietnam

**Keywords:** Civil engineering, Computer science

## Abstract

Vietnam, classified as a developing nation, encounters numerous challenges within its construction sector, including the scarcity of comprehensive and documented historical data regarding risks and a deficiency in embracing contemporary methodologies to mitigate the impact of risk factors on construction project objectives. This paper outlines initial findings from an ongoing research endeavor that centers on implementing Lean Construction (LC) techniques to enhance construction management practices specifically for marble floor finishing work within Vietnam. Therefore, this study aims to apply the construction lean principle combined with discrete-event simulation (DES) by using EZStrobe to simulate the marble floor finishing process in reality, from observing and collecting data of each activity in the actual process on the site. By building, running simulations, and resulting from real-world simulations, we'll understand the sources of waste, and then apply lean construction principles through methods such as just in time, reduce the batch size and resources priorities, and multi-skilled teams for the initial construction process. The study's lean modeling results has led to a 13% reduction in construction cycle time, a 141% improvement in process efficiency, a 268% enhancement in average productivity, and a 96% reduction in labor cost. The result has become the reference document resource for the managers and construction engineers to improve the performance of not only general finishing work but also marble floor finishing work.

## Introduction

Over the past two decades, the industry has improved significantly when applying many methods in operation and management and leading to many positive results in production such as productivity, efficiency, etc. The construction industry is also performing differently as operation and management methods improve slowly in the current information explosion era. Projects with large scale, complexity and high requirements appear more and more, besides, the project performance time is short in order to quickly put the product into use. Therefore, the construction industry faces recurring challenges such as cost overruns, time delays, low productivity, safety and quality issues, and excessive waste generation, which diminish customer value^1–3^. One of the another most common, unresolved and difficult challenges associated with the construction industry is the high rate of waste generation. Numerous studies in the construction industry^4–7^ have recently focused on waste generation during the construction life cycle.

Recently, there are quite lot of researches utilizing the Artificial Intelligence in construction field that was shown the superior of AI to solve the real industry problems. Son and Nguyen Dang^8^ presented the MVO model as an effective tool for addressing time–cost optimization issues in construction project management, surpassing other techniques in small-scale applications. Son and Nguyen Dang^9^ showcased a composite model named the hybrid multi-verse optimizer (hDMVO), which integrates MVO and the sine cosine algorithm (SCA). For repeating tasks with several concurrent instances, the adaptive selection slime mould algorithm (ASSMA) is presented by Son and Khoi^10^. Son and Khoi^11^ provides the mutation-crossover slime mold algorithm (MCSMA) for balancing time, cost, quality, and work continuity in a specific building project. In Vietnam, there has been extensive research in the field of artificial intelligence. Son and Nam^12^ employed Hybrid Sine Cosine Optimization to solve the Transport Project. Hybrid multi-verse optimizer model for a sizable discrete time–cost trade-off problem, by PVH Son and NDN Trinh^9^. To address the drawbacks of the GWO algorithm, Son and Trang^13^ introduces HDGM, an unique hybrid optimization model combining the dragonfly algorithm and the grey wolf optimizer. Weka software is described in Son and Luan^14^ and aids in modeling with strong algorithms and great dependability. Vu-Hong-Son et al.^15^ proposed project schedule optimization according to limited resources using a dependency structure matrix and whale optimization algorithm. Son and Lien^16^ seek to advance a Blockchain-based approach for meeting small- and medium-sized project delay resolution demands in a prompt and transparent manner.

Beside that one of the adaptive approaches addressing this issue, while not entirely unfamiliar, is the emergence of “lean construction” in the 1990s. This methodology seeks to implement lean production principles derived from the Toyota Production System (TPS) into the construction sector, with a focus on reducing waste and amplifying customer value^6,17,18^. Lean production practices aim to optimize project performance by minimizing waste and enhancing customer value^19,20^. The application of lean production methodologies is geared towards enhancing project efficiency by minimizing waste and elevating the importance of customer value^19,20^. Lean production has shown success in reducing waste by 12% in the manufacturing industry, while the construction industry has a significantly higher waste rate of 57%^21^. Wang et al.^22^ implemented the principles of flow production and lean construction within the framework of pipe spool shop fabrication, leading to notable enhancements in production performance. Abbasian-Hosseini et al.^3^ conducted an assessment of the advantages of lean construction by employing simulation techniques in a bricklaying process. The findings revealed substantial improvements: a 27% augmentation in operational efficiency, a 41% reduction in cycle time, and a notable 43% increase in productivity. Bamana et al.^23^ investigated the applicability of just-in-time, a pivotal component of lean construction, within wood construction, employing simulation techniques. The optimal scenario resulted in a reduction of total construction time from 26.09 to 22.31 weeks, concurrently mitigating downtime risks and amplifying the utilization rate of workers. This study aims to improve the construction process by implementing lean principles based on waste reduction. By evaluating performance factors in the construction process and applying lean thinking, the study will test and analyze lean principles using a case study and computer simulation. In practical application, it is evident that the testing of an innovative construction method incurs significant expenses and demands a substantial investment of time. Therefore, to achieve a thorough understanding of how lean thinking might impact the effectiveness of construction projects, a simulation-based methodology was utilized. Computer modeling and simulation precede real-world implementation for two primary purposes: to illuminate and quantify various forms of waste in the construction process and to identify and assess potential enhancements to the system's performance with minimal risk, cost, and time investment^24–26^. Moreover, simulation modeling is a cost-effective, flexible, accurate, and realistic approach, preferred by researchers due to its advantages over mathematical and experimental modeling^3,5,27,28^. For instance, Wang et al.^22^ implemented the principles of flow production and lean construction within the framework of pipe spool shop fabrication, leading to notable enhancements in production performance. Finally, the present study endeavors to compare the performance factors between the real-world model and the lean model, thereby offering valuable insights into the prospective efficacy of lean principles in waste mitigation during the construction process in improving performance.

## Literature review

### Lean construction

Lean construction does not exist in isolation; rather, it amalgamates concepts and principles originating from various sectors and subsequently adapted to the construction industry. Ohno^29^ bases its fundamental principles on lean manufacturing, which was originally conceived within the Toyota Production System (TPS) in Japan during the 1950s. Koskela^4^, recognized as a pioneer in the field of lean construction (LC), introduced this concept in a seminal paper from 1992, demonstrating the adaptation and implementation of lean manufacturing principles specifically tailored for the construction industry. Koskela et al.^30^ defines LC as a novel approach to production aimed at reducing time, resources, and effort to yield optimal results concerning value delivery. At its core, LC is viewed as a managerial philosophy that prioritizes tailoring customer value by minimizing various types of waste and establishing a seamless workflow integrated with ongoing enhancements, all underpinned by the principle of respecting individuals^31,32^. Glenn Ballard and Greg Howell established the Lean Construction Institute (LCI) in 1997, aiming to create and disseminate fresh perspectives on project management practices. According to LCI, the fundamental principles of LC involve optimizing entire processes, eliminating wasteful practices, emphasizing the workflow and its efficiency, creating value, and perpetually enhancing operations. Within the publication “Lean Construction: Core Concepts and New Frontiers,” various scholars, highly influential in the progression and development of LC, present a spectrum of concepts intertwined with these fundamental principles. They summarize the theoretical foundations, various lean construction methodologies, and the emerging possibilities within LC^33,34^.

Concerning the practical implementation of Lean Construction (LC), a multitude of tools and methodologies have emerged. Key lean methodologies encompass the last planner system, the pull-planning concept, and the lean project delivery method^32,35,36^. Mesa et al.^37^ introduced the lean project delivery system (LPDS), which implements lean principles and practices into project management and delivery procedures. LPDS strives to maximize stakeholder value by reducing waste, promoting collaboration, improving productivity, and optimizing project results^37^. Ballard^38^ emphasizes that the last planner system operates as a unified system wherein every component holds significant importance in facilitating lean project planning and execution. Fragmenting the system or selectively employing specific elements should be avoided^38^. Sarhan and Fox^39^ present compelling evidence supporting the application of lean principles in construction, illustrating numerous advantages derived from embracing lean thinking. These advantages include elevated productivity, enhanced reliability, heightened quality, improved client satisfaction, greater predictability, shortened project schedules, waste reduction, cost efficiency, improved feasibility in design, and upgraded safety protocols^40,41^. In Vietnam, there has been extensive research in the field of lean construction. Nguyen^42^ Evaluating outlook for lean construction in Vietnam through a study on perception of waste.

### Analysis of waste during construction on the site

Waste in the construction industry is categorized based on various characteristics, including type and quantity. While there are different classification systems, they generally share a common underlying concept. Among these classifications, the loss of materials, damaged products, and design flaws are commonly observed. For instance, Koskela^4^ identifies waste groups in construction, such as defects, rework, design errors, omissions, alterations, safety costs, and excessive material consumption. In addition to addressing these commonly known forms of waste, lean construction thinking focuses on identifying construction activities that consume worker time and resources without adding value to the final product. These activities, known as non-value-adding or value-free activities according to lean thinking, include duplication, transportation, unnecessary and excessive inventory, and worker waiting times. This study aims to measure and reduce waste during construction by applying lean construction principles, specifically targeting activities such as duplication, waiting, transportation, and unnecessary inventory.

### Computer simulation and lean construction

The practical implementation of new methods that lack prior testing often poses challenges in convincing contractors to adopt them. Moreover, historical evidence indicates that the construction industry tends to resist change^43^. Similar obstacles arise when attempting to apply lean thinking to construction processes. Computer simulations offer an ideal platform to explore the application of lean principles, analyze their impact, and develop a deeper comprehension of their functionality. By conducting virtual experiments, decision-makers can gain valuable insights into problem dynamics, benefiting from efficient and controlled trials without the costs associated with real-world trial and error^6,44^. Computer simulation encompasses the generation of a meticulously structured and coherent representation of an actual system existing in the real world, guided by mathematical and logical principles^43^. Through simulation, real-world processes can be effectively modeled and tested from an applied perspective. Consequently, this study utilized the combination of lean construction principles and computer simulation to address the identified problem before actual implementation at the construction site.

### EZStrobe software

As computer science continues to advance in the realm of graphic technology, there is an emerging inclination to employ graphical methodologies for the development of models and simulation of processes. Certain simulation software, widely adopted by construction researchers, includes STROBOSCOPE^27^, CYCLONE^45^, Extend™^46^, Extend þ BPR®^5^, SIMPHONY structure^22^, and WITNESS^47^. For this study, the EZStrobe software was employed to simulate the construction process of marble flooring. EZStrobe is a discrete event simulation system that employs Stroboscope's simulation engine and adopts the simulation model of three-phase active scanning. The EZStrobe simulation model is represented graphically through a network, with nodes and links easily constructed using drag-and-drop graphics from the EZStrobe template. This facilitates implementation without requiring significant effort. The EZStrobe model encompasses the various activities and resources involved in a construction project. EZStrobe enables modeling of moderately complex systems without the need for advanced computer coding^27^. Furthermore, the flexibility to swiftly modify the modeled process in response to changes in the real-world construction process is advantageous.

## Research methodology

**Figure 1:**
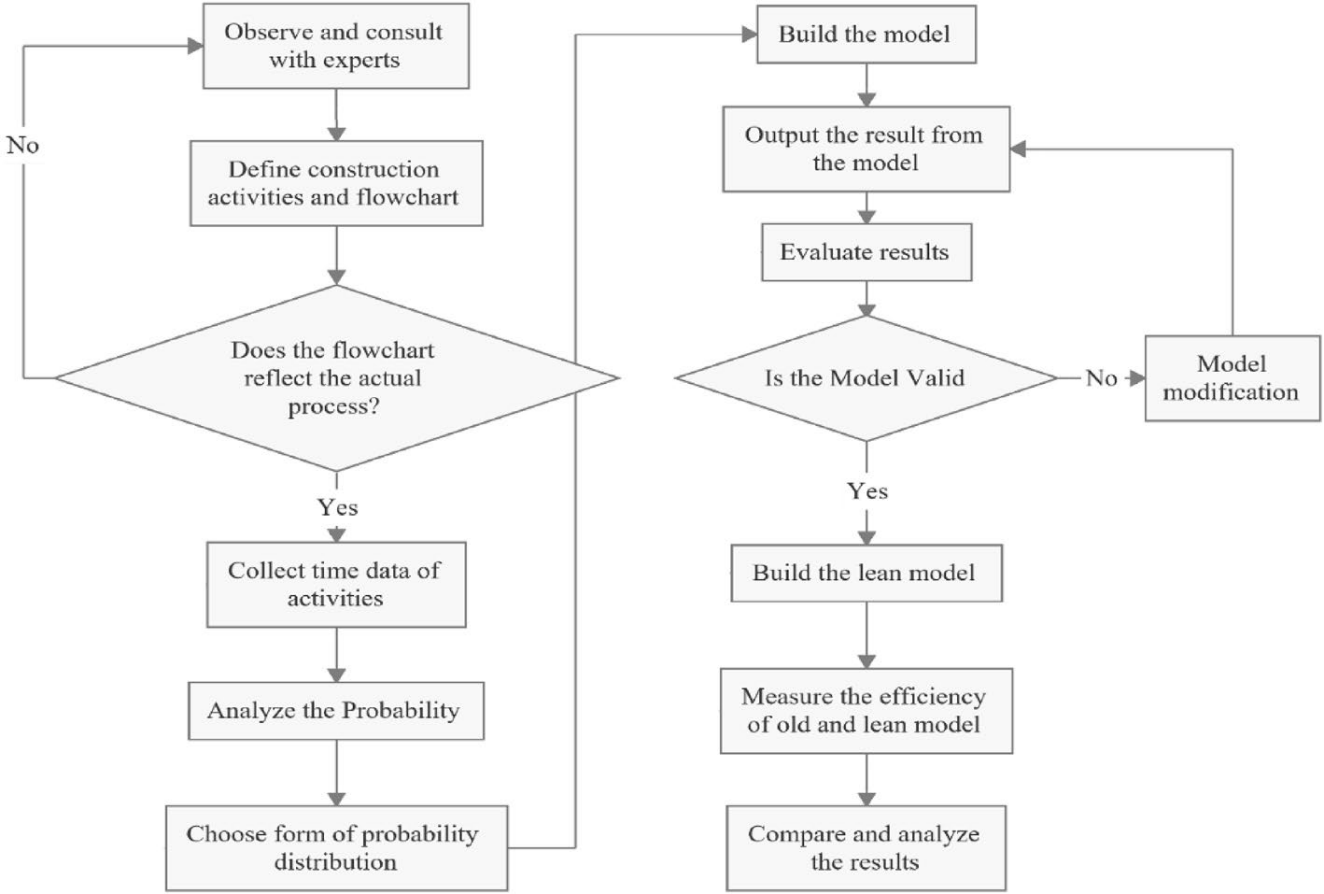
Research flowchart.

This section outlines the organization of the remaining study. Figure 1 illustrates the research process flowchart utilized to develop a reliable simulation model for analyzing the application of lean principles. Initially, an accurate understanding of the construction process is essential for simulating it effectively. This is achieved through meticulous observation, process mapping, and expert discussions. The second step involves data collection during construction to determine the probability distribution function for task durations. Subsequently, a computer simulation model is constructed based on the construction process map and the probability distribution function. The fourth step involves verifying and validating the developed model against real-world data to ensure its accuracy. Any necessary adjustments are made to address discrepancies between the model and reality. Once validated, the real-world model is modified to incorporate the chosen lean principles, resulting in a new model referred to as the lean model. Subsequently, the evaluation of the real-world and lean models is conducted by quantifying their performance indicators such as cycle time, productivity, process efficiency and labor cost. Finally, a comparison between the outputs of the two models is conducted to assess the applicability of lean concepts in the construction process.

Construction managers often rely on intuition or experience to make decisions, but this approach is not the most efficient for the construction process. Additionally, it is costly and time-consuming to adjust existing processes. Hence, crafting and enhancing construction procedures, particularly with innovative approaches such as lean construction, can pose considerable challenges. Computer simulation emerges as a potent remedy, offering a controlled and cost-efficient setting for decision-makers to design, assess, and refine processes through experimental iterations. Simulation also enhances understanding of workflow in construction processes. Thus, a simulation-based approach was adopted in this study to thoroughly understand the potential impacts of lean principles on construction project performance. Preceding real-world implementation, computer-based modeling and simulation are employed for dual objectives: (1) the identification and quantification of diverse waste categories encountered during the construction process, and (2) the examination and assessment of prospective enhancements in performance, time, and cost. Simulation modeling is deemed advantageous due to its cost-effectiveness, adaptability, precision, and fidelity when compared to mathematical and experimental modeling approaches. In this study, discrete event simulation (DES) was chosen to achieve the study's objectives.

Following the research process depicted in Fig. 1, a field trial was conducted to test and evaluate the effectiveness of lean construction principles in reducing waste in the marble floor finishing construction of a showroom and model apartment with a 10,000 m2 floor area. The marble floor construction process consists of interconnected activities and resources, which are time-consuming and challenging. This cyclical nature makes it suitable for lean evaluation through computer simulation. The study demonstrates the results of applying the five lean principles (defining value, mapping the value stream, creating flow, using a pull system, and pursuing perfection) along with conventional simulation modeling steps (data collection, activity duration fitting, model testing/validation/verification, and model improvement).

The suggested approach initiates with a field investigation grounded in two foundational lean construction principles: delineating value and charting the value stream. These principles endeavor to comprehend the framework and rationale behind marble floor finishing construction tasks, intending to formulate a comprehensive map illustrating the value stream within the present on-site construction process. This visual representation aids in the identification of prevailing challenges and facilitates informed decision-making concerning prospective enhancements to the system's performance. Data on each activity in the 10,000 m^2^ floor area were meticulously collected. software's Batch Fit function, whereby the most dependable distributions were chosen through fit-quality assessments. Upon confirming the congruity between the observed marble floor construction process and the actual process, a real-world simulation model was constructed utilizing the EZStrobe simulation software.

The validation and verification procedures were undertaken to refine the model and enhance its alignment with the operational dynamics of the examined process. Multiple replications were executed to establish a validated and reliable real-world simulation model. Subsequently, three lean construction principles were proposed and tested: just-in-time (JIT), reduction of batch size and resource priorities, and multi-skilled teams. These principles resulted in an improved model (lean simulation model). The performance metrics, including process efficiency, labor productivity, cycle time, and labor cost, were compared between the real-world model and the lean model to evaluate the effectiveness of lean construction principles in improving the performance of the construction process.

## Case study

**Figure 2:**
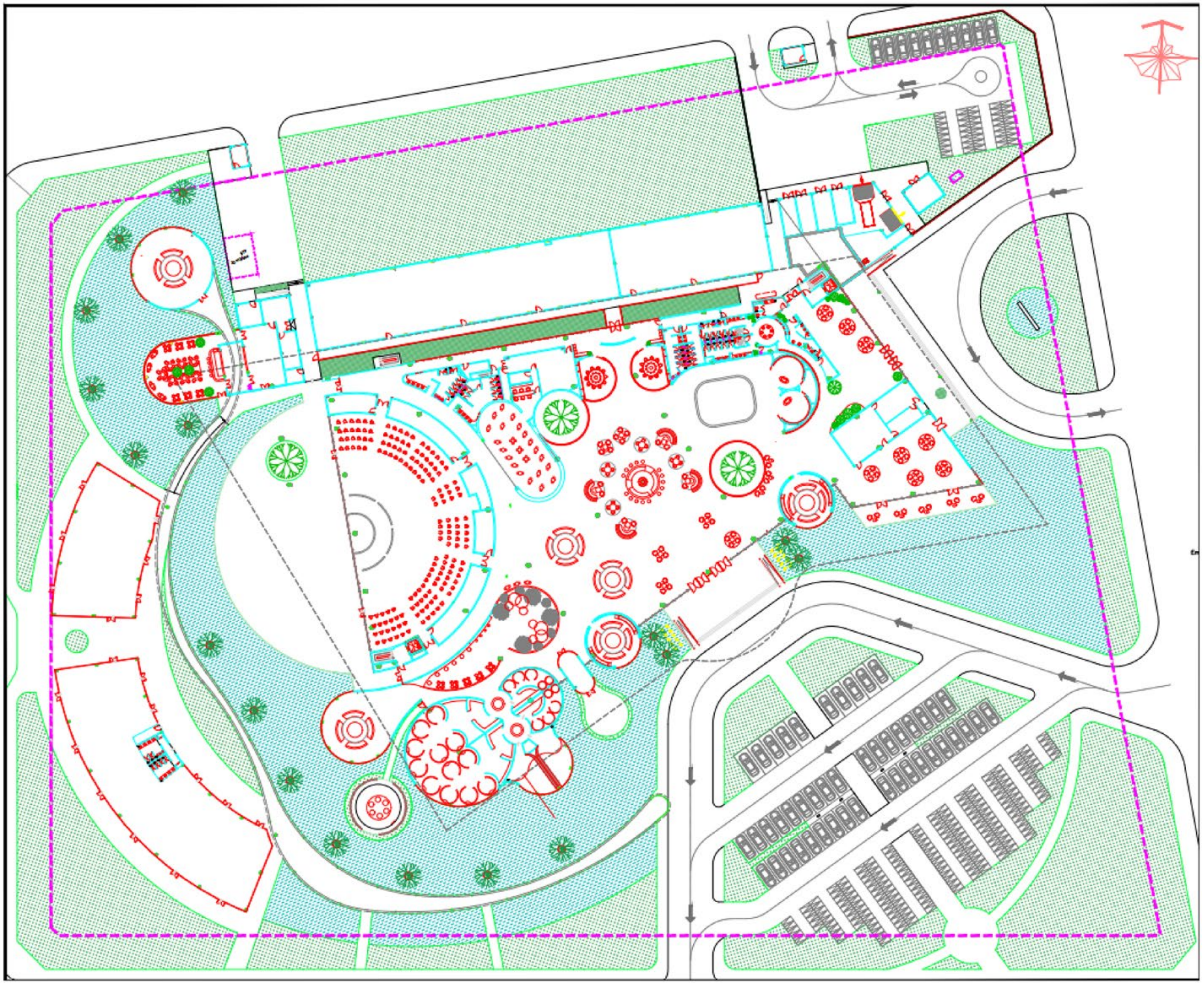
Total construction site.

In order to examine and assess the suitability of lean principles in construction processes, the implementation of a practical experiment becomes essential. Hence, this research endeavors to extend the exploration of lean feasibility in construction by stone floor finishing operation of a sale gallery and sample apartment project (see Fig. 2). A subcontractor is responsible for the completion of the paving work, including paving for all 10,000 m^2^ of floor. All marble floors are on the same level. Based on the fundamental timeline, the contractor is anticipated to finalize the work of the floor stone within a period of 120 days.

### Building actual construction flowchart and collecting data

**Figure 3:**
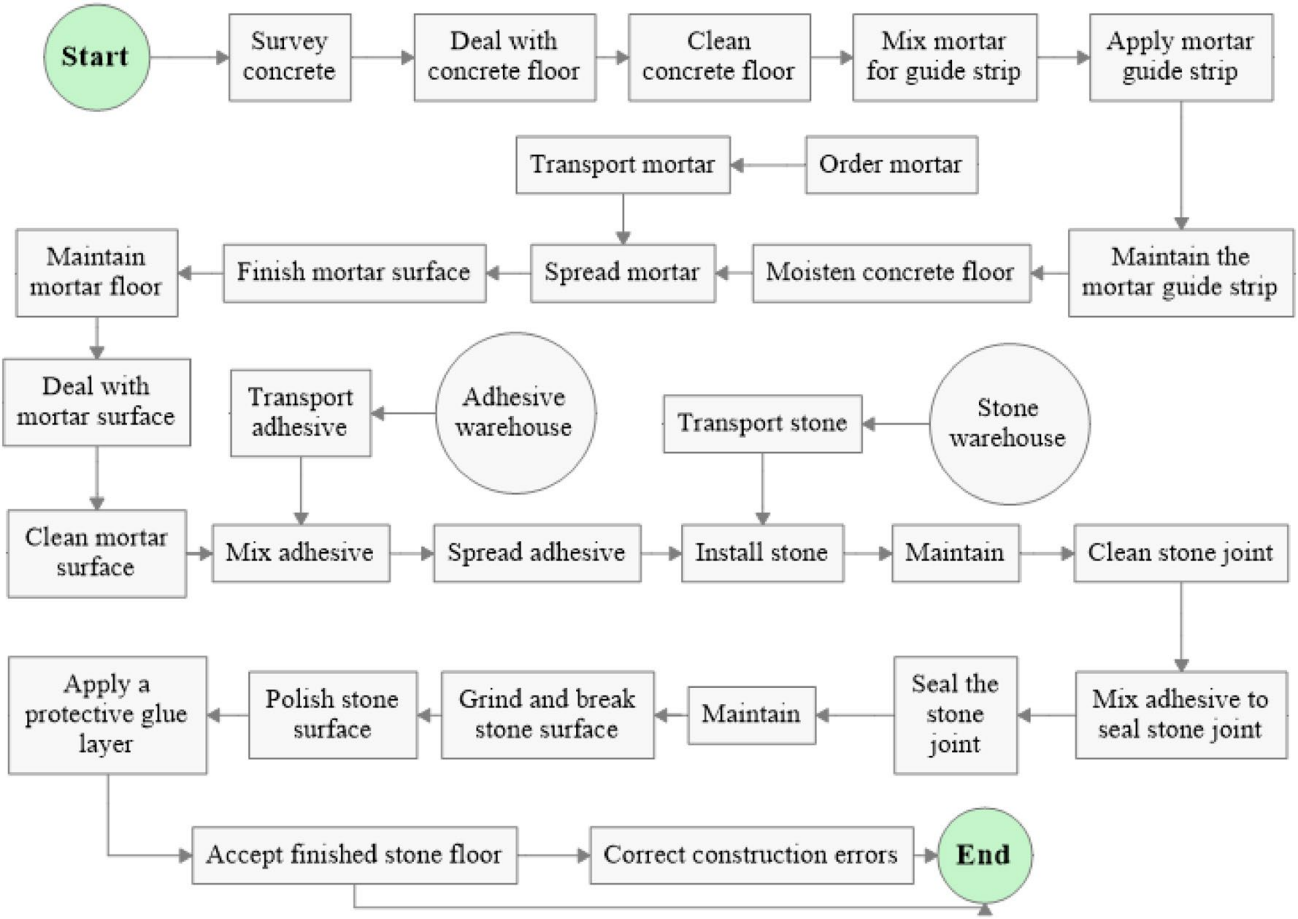
Process map of marble floor finishing work.

The initial step in constructing a simulation model involves defining process activities, their sequence, labor and resources, and workflow based on the construction process on site. As Al-Sudairi^5^ explains, the interdependencies among resources, activities, connections, and material or information flow within any construction process can be illustrated through the utilization of a “process flowchart”. This flowchart visually represents the workflow and defines the value stream, and it is created based on field observations and expert discussions. The primary process flowchart is initially formulated based on the process layout and subsequently enhanced and verified through in-depth deliberations with domain experts. It is important to note that a flowchart serves not only as an efficient tool for developing the simulation model but also as a key element in the application of lean principles, as it provides a visual representation of the workflow. Within the context of this study, the configuration of the marble floor finishing process was derived through firsthand observation and the utilization of video recording techniques (see Fig. 3). The study focused on groups of workers, including main workers and sub-workers. The mason group carries out the floor mortar activities, the stone mason group performs the paving activities, and the grinder group performs the marble floor finishing activities.

The simulation model employs a stochastic time interval for each activity, necessitating the selection of values from a specific dataset. Therefore, after completing the process map, the next step is to define quantitative data for each task, which is the input to the simulation model. Conducting detailed and comprehensive fieldwork through time engineering and research work to collect this quantitative data. These methodologies rely on capturing the complete process of marble floor finishing through video recording and subsequently quantifying the duration of each individual activity. The typical approach to modeling a stochastic process involves the identification and calibration of a probability distribution that best aligns with the constituent elements of said process, leveraging sample data as the foundation for such selection and fitting. Empirical data only provides values based on observed occurrences, while it may lack the ability to generate a comprehensive range of values. Additionally, anomalies within the empirical distribution can be identified^5^. Hence, it is recommended to employ appropriate data distribution functions instead of relying solely on empirical distributions for simulation. Various software options are available to match the sample data distribution, making this process fast, effortless, and accurate^5^.

Crystal Ball, the software utilized in this study, is well-suited for accommodating a wide range of distributions for sampled observations due to its user-friendly attributes. As indicated on its website, The Crystal Ball software is a readily accessible simulation tool created to examine the uncertainties and risks connected to models within Microsoft Excel spreadsheets. Functioning as a seamlessly integrated Excel add-in tool with a dedicated toolbar and menu options, Crystal Ball expands spreadsheet functionalities by accommodating diverse distributions for sampled observations. Prominent characteristics include correlation assessment, fitting distributions, a diverse distribution gallery, forecast charts, sensitivity analyses, precision management, Latin Hypercube sampling, and additional functionalities. The provision of charts and reports enables users to swiftly identify the optimal distribution for the specific activity. As an illustration, the process of identifying the optimal distribution for the specific activity. To do so, from the research technique in statistical probabilities 15 data points from 15 observations over the duration of the activity were required through the review of videotape recorded on site. The application of the Crystal Ball software, specifically utilizing the Batch Fit feature, encompasses a range of continuous distributions (such as Exponential, Beta, Gamma, Uniform, etc.) that have undergone testing against the gathered data. Subsequently, the most favorable distributions, as determined by fit tests such as Chi-square, Anderson Darling, and Kolmogorov–Smirnov, were selected to validate the intended distribution.

### Building simulation model

**Figure 4:**
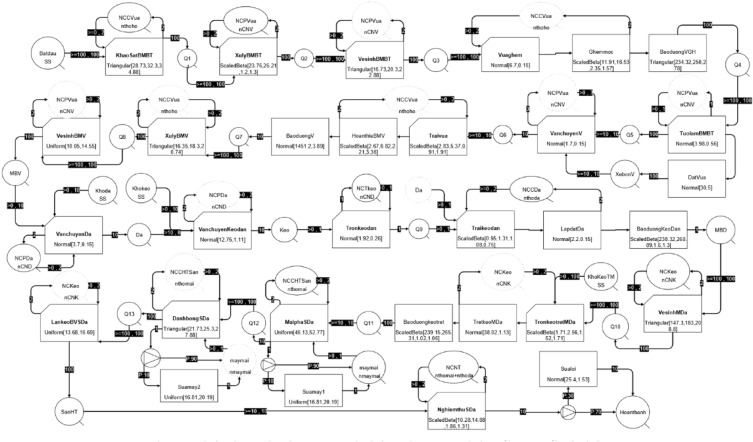
Real-world simulation model in the marble floor finishing process.

After finding the distribution patterns for the most suitable activities, simulations were developed for the construction process of marble floor finishing. The EZStrobe simulation software utilizes process maps, distribution parameters, and empirical behavioral observations as inputs to precisely model typical construction processes (see Fig. 4). The model is composed of diverse modules that strive to closely replicate real-time events by incorporating a system of conditional jobs, routines, and queues, thereby advancing its fidelity to the actual occurrences. Activities will be assigned time and cost, thereby optimizing the process to reduce waste and improve process efficiency.

### Verifying and validating the simulation model

The efficacy of the modeling process is evident when the simulation model faithfully depicts the established workflow and interconnections among different tasks. Therefore, before embarking on any experimentation involving simulated models, it is imperative to undertake thorough testing and validation of these models^27^. The process of testing guarantees that the model operates according to its intended purpose and is devoid of any logical inconsistencies or errors^5^. In practice, testing involves examining the computer code, running tests, and assessing the statistical consistency of the model's formal representation^48^. On the other hand, model validation guarantees the fidelity of the simulated model in mirroring the authentic behavior of the process^5^. During the validation step, the cycle time of the model, obtained from test runs, is compared with the cycle time of the real-world system to evaluate the realism of the model assumptions. Validation serves as a crucial phase in developing a dependable simulation model^48^. To achieve a close alignment between the simulated model and the actual behavior, it is crucial to conduct multiple replications, resulting in a verified and validated model.

#### Testing simulation model

Model testing encompasses activities such as validating logical consistency within the model, conducting simulation tests, monitoring entities along sample path trajectories, and assessing the coherence of results^48^. In the context of construction process simulation testing, the activities were carefully examined to ensure their proper execution and adherence to predefined conditions. Likewise, all activities, modules, links, and resources underwent thorough testing and verification to ensure their accurate functionality.

#### Validating the simulation model

As previously mentioned, the conventional approach to validation involves comparing real-world data with the outputs generated by the simulation model^48^. The evaluation of cycle time serves as a crucial factor in assessing the resemblance between a real-world process and a simulated process. Moran et al.^49^ highlighted the significance of time as a valuable and universally applicable metric for comparisons, as it can lead to improvements in cost and quality. Cycle time comparisons have also been employed by previous researchers such as Al‐Sudairi^5^. Hassan and Gruber^27^, and Wang et al.^22^ to validate their respective models. Hence, in this study, the validation process involved conducting cycle time comparisons.

**Figure 5:**
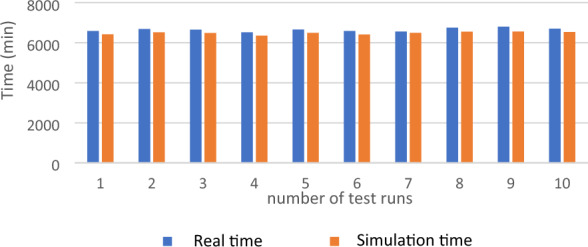
Validation of the real-world simulation model.

Following each test, necessary adjustments were made to align the simulation model more closely with the actual construction process. To carry out model validation effectively, it was crucial to determine the number of simulations runs required to achieve the desired level of accuracy. In general, a single run of the model is insufficient for producing consistent output^27^. Yeh and Schmeiser^50^ proposed that conducting ten to thirty replications is sufficient to achieve a desirable level of accuracy. Similarly, Hassan and Gruber^27^ established the validation of their model through the utilization of ten replications. Hence, in this study, ten replications were employed for model validation and other calculations. The difference between the mean of 10 field observations and 10 model runs is 2.62% less than 5%, which is considered acceptable^3,25,27,51,52^. The results are shown in Fig. 5.

### Applying lean construction principles and models

After the process model of real world marble floor finishing was developed, it was tested and validated and the results were obtained after computer simulation of the real world model. Consequently, the current stage necessitates the enhancement of the stone floor finishing process through the incorporation of lean construction principles. In accordance with this objective, three distinct lean construction approaches, specifically just-in-time (JIT), reduction of batch size and resource prioritization, and the utilization of multi-skilled teams, are implemented within the observed process.

#### Principles of production chain

**Figure 6:**
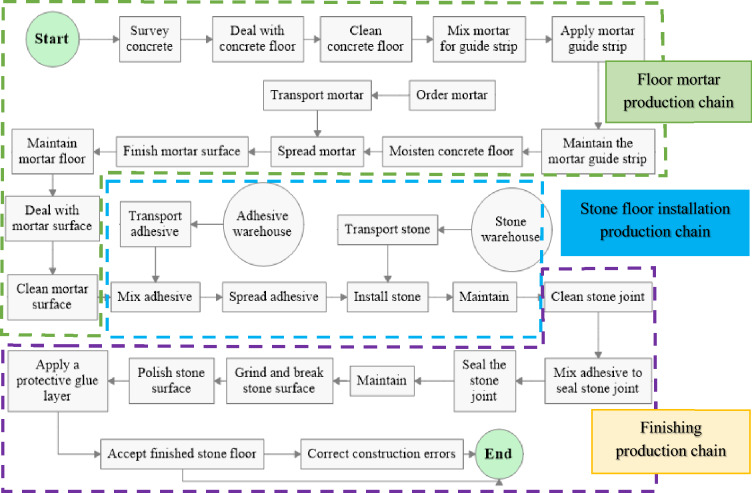
Production chain of stone floor finishing process.

The stone *floor* finishing process is divided into 03 production chains, including the floor mortar production chain, stone floor installation production chain, and stone floor finishing production chain which are the basis of the stone floor finishing process shown in the Fig. 6. These three production chains are interrelated in many respects, making the modeling process complicated. The process of mapping the stone floor construction process not only enhances the comprehension of the value stream leading to the final product, but also facilitates a deeper understanding of the interrelationships among activities, linkages, and resources. It is important to emphasize that this concept alone does not result in improvement, but rather complements and serves as a foundational element for the implementation of other lean principles and techniques.

#### Just in time

In the real world, stone and adhesive materials are delivered to the site early with a 1000 m^2^ lot which leads to storage and transportation of stone and adhesive from the warehouse to the construction site. The just-in-time method is used to maintain the flow of building materials in line with the site requirements. This means that stone and adhesive orders will be delivered to the 100 m^2^ lot directly to the construction site and the order will be carried out while waiting for the floor mortar construction. This means that ordering stone materials will replace stone and adhesive transportation in the real world model.

#### Reduce batch size and resources priorities

During the observation of the stone floor paving and finishing process, it was noted that tasks such as stone installation, floor grinding, and breaking ground were being accumulated in large batches before being transferred to the subsequent finishing step. This approach resulted in significant waiting times for workers, as they had to wait for a sufficient quantity of materials to proceed to the next construction phase. Consequently, this led to increased inventory, prolonged wait times, and reduced labor efficiency. Such a sequential approach hindered the simultaneous progress of entities involved in stone floor finishing, negatively impacting overall performance.

In line with the principles of lean production, the pull method emphasizes that downstream workstations should be direct consumers of materials or parts from upstream workstations, ensuring timely supply in the correct quantity. In order to address this issue, a real-world simulation was conducted, reducing the batch size of the stone floor finishing production chain from 100 to 10, as depicted in the illustration. This adjustment facilitated the concurrent execution of all marble floor finishing activities, including grinding, breaking, and polishing. Consequently, resource waiting times were minimized since activities no longer had to wait for a large ground area to be processed. Additionally, priority was given to the grinding and breaking activities, as they involved substantial wait times for the stone surface preparation and the grinding machine.

#### Multi-skilled team

As previously mentioned, the workers in the marble floor construction process are assigned to specific production chains based on their skills and capabilities. These production chains include the floor mortar production chain, stone surface production chain, and stone floor surface finishing production chain. For instance, masons and mortar workers are exclusively involved in activities within the mortar production chain, limiting their involvement in more critical tasks. Studies conducted by Al-Sudairi et al. (1999), Diekmann et al. (2004), and Esquenazi and Sacks (2006) have highlighted that the absence of multi-skilled workers in lean processes reduces flexibility and constrains productivity in construction^53–55^.

To address this issue, it is proposed to introduce a multi-skilled team capable of performing all activities in the marble floor construction process. This aligns with the principles of lean processes and aims to test the potential improvements related to waste reduction (e.g., waiting and inventory) and enhanced labor productivity through real-world simulations. In the improved model, resource allocation was modified, and only primary and secondary workers were retained, eliminating the need for specialized roles such as masons, mortar workers, stone masons, stone workers, grinders, and adhesive workers. This new approach enables continuous work for the workers without waiting for the next construction site. Importantly, the introduction of multi-skilled workers facilitates accelerated processing and production flow balance when value-adding activities such as floor mortar, stone installation, and stone finishing are performed. The availability of additional resources enhances processing efficiency and improves the overall workflow.

## Evaluation of lean model improvement

**Figure 7:**
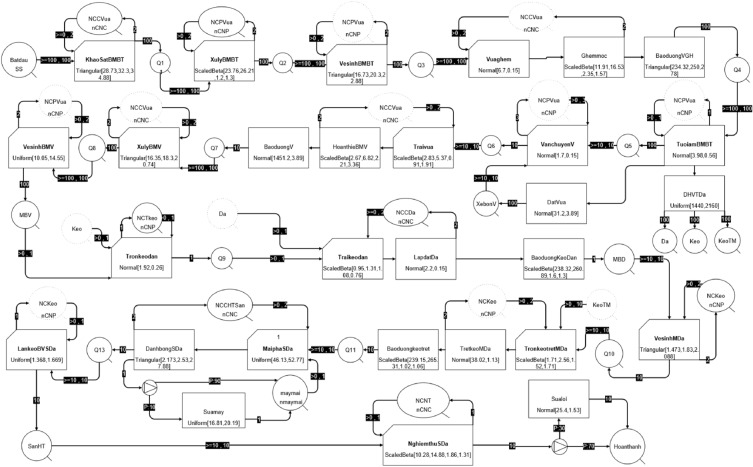
Lean simulation model of marble floor finishing process.

Once the lean principles have been implemented in the real-world model and transformed into a lean model (see Fig. 7), the next step involves assessing the potential impact of these principles on construction. This evaluation involves the examination and analysis of the simulation outcomes derived from both the real-world model and the lean model to assess the effectiveness of incorporating lean principles. Key factors such as process efficiency, labor productivity, cycle time, and labor cost are calculated and compared between the two models. This comparison enables a comprehensive assessment of the efficiency achieved through the application of lean principles.

### Process efficiency

Efficiency is a vital performance metric within a given process^56^. In the context of implementing a lean methodology, the measurement of process efficiency can be accomplished by juxtaposing the duration allocated to value-adding activities against the entirety of the cycle time^5^. In fact, it can be appropriately evaluated on the level of worker's work efficiency. In the present study, the quantification of process efficiency involves the computation of the ratio between the labor time utilized for value-adding activities and the total labor time expended throughout the construction process (Eq. 1).

1 $${\text{Process efficiency}} = \left( {{\text{Total labor time}} - {\text{Time spent on non}} - {\text{value adding activities}}} \right)/{\text{Total labor time}}$$

As Table 1 shows, this lean model's process efficiency is increased by 268% from eliminating and reducing the time spent on non-value adding activities such as transportation, storage, and wait time.

**Table 1:** Evaluation table of lean model improvement.

	Process efficiency	Productivity (m^2^/work)	Construction cycle time (h)	Labor cost (VND)
Real world model	0.136	3.53	107.83	182 million
Lean model	0.501	8.52	93.8	72.4 million
Improvement	268%	141%	13%	96%

### Labor productivity

As process flows are assessed with respect to time, productivity rates can serve as a straightforward means of measuring and comparing construction activities^57^. Productivity in terms of “input/output” considering labor as an input parameter is commonly used in the construction industry^58^ and therefore, it is used in this study.

By the nature of the construction process, worker performance is the main factor affecting productivity value. Labor productivity is measured by the number of square meters of actual working floor per unit of time. Therefore, Eq. (2) is as follows:

2 $${\text{Labor productivity}} = {\text{input}}/{\text{output}} = {\text{floor area}}/{\text{construction time}}$$

Labor productivity of the lean model is 8.52 m^2^/work, an improvement of 141% compared to 3.53 m^2^/work. Construction cycle time is reduced and the number of manpower required is low due to the flexibility of manpower leading to coordination between activities in the process, reducing worker wait times is a major factor in this result.

### Cycle time

Ensuring the timely completion of the construction process not only results in the timely accomplishment of a project but also has the potential to enhance labor productivity and process efficiency. As a result, evaluating the cycle time as a means of comparing alternative construction processes may be considered a fitting criterion for evaluation. The results are listed from Table 1, the construction cycle time is 93.8 h, down 13% compared to 107.83. The result gained is a faster process with no long wait times. Moreover, from the results, it can be seen that the floor finishing items can be completed faster than the original schedule.

### Labor cost

Besides time, cost is also the core evaluation factor in evaluating the improvement of lean processes to increase the persuasion for companies and experts in the construction industry. They largely determine the possibility of using a new method of construction. Therefore, Eq. (3) is as follows:

3 $$\begin{aligned} {\text{Labor cost}} & = {\text{number of main workers}}*{\text{workday}}*{\text{unit price}} \\ & \quad + {\text{number of auxiliary workers}}*{\text{workday}}*{\text{unit price}} \\ \end{aligned}$$

From the improvement of the above factors, the labor cost of the lean model is 72.4 million VND, down 96% compared to 182 million VND from the real world model. In addition, reducing the construction cycle time will further reduce indirect costs and bring more profits to the contractor.

## Conclusion

Industry has always struggled with inefficiencies in production processes. Similarly, the construction industry inevitably faces the challenge of improving efficiency by using fewer resources. A good example that can easily be seen is the change in operating costs by reducing waste, improving the efficiency, process performance which can create sustainable changes in terms of profits for construction companies and enterprises.

This research introduces a systematic methodology for implementing lean production principles in construction procedures, with a specific emphasis on waste reduction. The approach involves the creation and testing of a marble floor finishing process model through the utilization of EZStrobe simulation software. The study's findings substantiate the considerable potential of these principles in enhancing construction processes and minimizing the generation of waste within those processes. Three additional lean construction principles, namely “just in time,” “reduce batch size and prioritize resources,” and “multi-skilled teams,” were applied to the initial marble floor finishing process. The implementation of these principles yielded significant improvements, including a 13% reduction in cycle times, a 141% increase in productivity, a 268% enhancement in process efficiency, and a 96% reduction in labor costs. Importantly, the cost of implementing these methods proved to be more economical than the benefits derived from embracing lean construction principles. Moreover, by adopting lean construction methods, companies gain valuable insights into the various types of waste present in their construction processes. This tool facilitates the identification of root causes, enabling informed decision-making regarding areas in need of change. Consequently, it streamlines problem prioritization and mitigates sub-optimization. The utilization of lean construction techniques contributes to the overall efficiency of construction activities, resulting in reduced operating costs, increased profit margins, and minimized environmental impact.

This research is grounded in the analysis of data obtained from a specific marble floor finishing process, showcasing notable enhancements in performance based on simulation outcomes derived from a residential building endeavor in Vietnam. Consequently, the observed performance enhancements in this study are exclusively pertinent to projects executed within the Vietnamese context. To enhance the study, the incorporation of additional principles applicable to a diverse range of construction projects beyond residential buildings is recommended. Additionally, conducting an action research approach could gauge the efficacy of various metrics, including construction productivity, efficiency, cycle time, and labor in real projects. This approach aims to encourage practitioners to more frequently apply lean construction principles in their endeavors, thereby fully realizing the potential inherent in the lean construction philosophy.

## Data Availability

Some or all data, models, or code that support the findings of this study are available from the corresponding author upon reasonable request.
